# Sex Differences in Monocyte Activation in Systemic Lupus Erythematosus (SLE)

**DOI:** 10.1371/journal.pone.0114589

**Published:** 2014-12-08

**Authors:** Wei Jiang, Lumin Zhang, Ren Lang, Zihai Li, Gary Gilkeson

**Affiliations:** 1 Department of Microbiology and Immunology, Division of Infectious Diseases, Department of Medicine, Medical University of South Carolina, Charleston, SC, 29425, United States of America; 2 Department of Hepatobiliary Surgery, Beijing Chaoyang Hospital, Capital Medical University, Beijing, 100020, China; 3 Division of Rheumatology, Department of Medicine, Medical University of South Carolina, Charleston, SC, 29425, United States of America; Beth Israel Deaconess Medical Center, United States of America

## Abstract

**Introduction:**

TLR7/8 and TLR9 signaling pathways have been extensively studied in systemic lupus erythematosus (SLE) as possible mediators of disease. Monocytes are a major source of pro-inflammatory cytokines and are understudied in SLE. In the current project, we investigated sex differences in monocyte activation and its implications in SLE disease pathogenesis.

**Methods:**

Human blood samples from 27 healthy male controls, 32 healthy female controls, and 25 female patients with SLE matched for age and race were studied. Monocyte activation was tested by flow cytometry and ELISA, including subset proportions, CD14, CD80 and CD86 expression, the percentage of IL-6-producing monocytes, plasma levels of sCD14 and IL-6, and urine levels of creatinine.

**Results:**

Monocytes were significantly more activated in women compared to men and in patients with SLE compared to controls *in vivo*. We observed increased proportions of non-classic monocytes, decreased proportions of classic monocytes, elevated levels of plasma sCD14 as well as reduced surface expression of CD14 on monocytes comparing women to men and lupus patients to controls. Plasma levels of IL-6 were positively related to sCD14 and serum creatinine.

**Conclusion:**

Monocyte activation and TLR4 responsiveness are altered in women compared to men and in patients with SLE compared to controls. These sex differences may allow persistent systemic inflammation and resultant enhanced SLE susceptibility.

## Introduction

Women exhibit differences in Toll-like receptor (TLR) 7 responsiveness, T regulatory cell activity, and environmental factor exposure compared to men [1]–[10]. These differences may account for the stronger cellular and humoral immune responses in women, as well as their higher risk of autoimmune diseases [8]. Systemic lupus erythematosus (SLE) occurs primarily in women at a ratio of 9∶1 compared to men [2]. Although host immune factors, epigenetic and environmental factors may partially account for the higher prevalence of SLE in women, the exact mechanisms are not fully understood. The onset of SLE disease most often occurs in women during the child-bearing years, therefore sex hormones are believed to play a major role in the etiology of SLE disease [11].

In the periphery, plasmacytoid dendritic cells (pDCs), as well as other immune cells, express estrogen receptor alpha (ERα) [12], pDCs play an important role in SLE disease pathogenesis due to their function in mediating immune responses as well as their producing large amounts of IFN-α in response to TLR7 and TLR9 ligands [13], [14] Knockout of ERα in both control and lupus prone mouse strains resulted in reduced TLR3, TLR4, TLR7 and TLR9 responses in pDCs, spleen cells and B cells [15], suggesting that estrogen signaling affects TLR responsiveness. Guery's group showed that pre-menopausal, not post-menopausal women, have increased pDC responses to TLR ligands compared to men through a cell-intrinsic ERα signaling [16], [17]. Being located on the X chromosome, TLR7 responsiveness, as shown in IFN-α production, in pDCs from women is higher than men [7], [18]. Given that women possess have two TLR7 genes, compared to the one in men, led to speculation that epigenetic factors/X chromosome inactivation issues may partially explain enhanced female responsiveness to TLR7 agonists. Treatments targeting TLR7/8 and TLR9 are in Phase I trials in patients with SLE and should provide insight into the role of TLR signaling in lupus including whether these therapies will be more effective in women than men [19]–[22]. Other immune cells have variable ER expression. B cells express ERβ, CD4 T cells express ERα, CD8 T cells and monocytes may express low levels of both ERs [12]. Previous studies have reported that TLR responses in certain cell types (e.g., DCs and macrophages) are modulated by estrogen [17], [23]. ER expression was analyzed by qPCR only, due to the lack of reliable antibodies for analyzing protein expression by western blots or flow cytometry. Importantly, these assays were done in humanized mice, cell lines, or *in vitro* in activated primary cells, but have not been proved in freshly isolated DCs, monocytes, or macrophages in human *ex vivo*. Whether sex hormones have a direct or indirect effect on TLR signaling pathway in these immune cells is not clear as ERα expression impacts development of a number of different cell types.

Monocytes are divided into three subsets [24]–[28], classic monocytes (CD14++CD16-), intermediated monocytes (CD14++CD16+), and non-classic monocytes (CD14+CD16++). Classic monocytes produce IL-10 in response to the TLR4 ligand LPS; non-classic monocytes produce TNF-α and IL-1β in response to TLR7/8 ligands [29], [30]. Non-classic monocytes respond to viral stimuli and immune complexes via a TLR7 or TLR8 pathway [26], and play a role in cardiovascular diseases [27]. Heightened CD16 expression occurs in inflammatory conditions, such as sepsis [31], HIV disease [27], atherosclerosis [24], [27], [32], cancer [33], and autoimmune diseases (e.g., rheumatoid arthritis, SLE) [34]–[36], suggesting that inflammation (including TLR ligands) could be the drivers for monocyte differentiation into a CD16-expressing subset *in vivo*. The data on monocyte ER expression is controversial [12], [37]–[40]. Whether estradiol regulates CD16 expression on monocytes is not clear [41], [42]. Human monocytes express high levels of TLR4 and are the primary cells in the periphery that respond to the TLR4 ligand LPS [43]. They produce large amounts of pro-inflammatory cytokines (e.g., IL-6, TNF-α, IL-1β and sCD14) and express activation molecules (e.g., CD80, CD86) in response to LPS stimulation [14], [44], [45]. Monocytes can also differentiate into macrophages and DCs under certain conditions [46]–[48].

In the current project, human primary monocytes were studied from healthy male and female controls and female patients with SLE. We found that purified monocytes from healthy controls produced sCD14 in response to LPS *in vitro*. Healthy women had higher levels of plasma sCD14 and lower levels of surface CD14 on monocytes compared to healthy men *in vivo* or *ex vivo*. Healthy women also had elevated numbers of non-classic monocytes that expressed higher levels of CD86, and reduced proportions of classic monocytes compared to age and race matched males. In patients with SLE, female patients had further enhanced monocyte activation compared to healthy control women.

## Materials and Methods

### Study subjects

This study was approved by the Institutional Review Board for Human Research (IRB) at the Medical University of South Carolina. All subjects were adults ages 20 to 50 and provided written informed consent. In the present study, 27 healthy male controls, 32 healthy female controls, and 25 female patients with SLE were enrolled. Groups were matched for age and race. Due to our patient population, the majority of the participants were African American. Controls were free from any symptoms or signs of an autoimmune disease. SLE patients all fit the diagnostic criteria for systemic lupus as published by the American College of Rheumatology [49]. Disease activity in lupus patients was assessed by the SLE disease Activity Index 2000 (SLEDAI) by the treating rheumatologist who is trained and experienced using the SLEDAI [50]. Disease damage was calculated by the SLICC DI (Systemic Lupus Erythematosus International Collaborating Clinics Damage Index), again being performed by the treating rheumatologist familiar with the instrument [51]. 5% female controls and 28% female patients are under hormone treatments, 25% female controls and 20% female patients are post-menopausal women.

### Cells

Whole blood was collected in heparin-coated tubes, and peripheral blood mononuclear cells (PBMCs) were isolated over a Ficoll-Hypaque cushion. Purified monocytes were obtained from PBMCs by negative selection (Miltenyi Biotec, Bergisch Gladbach, Germany); the purity was above 90%. Monocytes (1×10^6^ cells/mL) were cultured with or without LPS (0.5, 5 or 50 ng/mL, Sigma, St. Louis, MO) for 5 h, cell culture supernatants were harvested and stored at −80°C for sCD14 testing.

### Cell surface and intracellular staining

For surface staining, antibodies were incubated with whole blood or PBMCs at room temperature for 10 minutes. After surface staining, the red cells were lysed, or the PBMCs were washed and stained intracellularly using Perm/fix reagents (BD) according to the manufacturer's protocol. After intracellular staining, cells were immediately analyzed by flow cytometry.

### Flow cytometry

The fluorochrome-labeled monoclonal antibodies used in this study included: antibodies against CD14-percp (BD), CD16-FITC (BD Pharmingen), IL-6-phycoerytherin (PE, BD Pharmingen), CD80-allophycocyanin (APC, BD Pharmingen), CD86-PE, and isotype control antibodes (BD Pharmingen). Cells were identified by their forward and side scatter characteristics and were analyzed by flow cytometry on a Guava 8HT flow cytometer (Millipore, Billerica, MA).

### Plasma levels of IL-6 and soluble CD14

Plasma samples were collected into tubes containing EDTA and after centrifugation were stored at −80°C until they were thawed for analysis of sCD14 and IL-6. Plasma levels of sCD14 and IL-6 were quantified using a commercial kit according to the manufacturer's protocol (R & D, Minneapolis, MN).

### Statistical analysis

The differences in continuous measurements between the groups were compared by the Mann-Whitney's *U* test. To explore associations between pairs of continuous variables, Spearman's rank correlation was used. Comparison analysis was performed using SPSS software (version 16.01). All tests were 2-sided, and P≤0.05 was considered to be statistically significance.

## Results

### Increased proportions of non-classic monocytes and decreased proportions of classic monocytes in healthy women compared to healthy men

Previous studies have delineated that three monocyte subsets have distinguishable responsiveness to bacterial and viral products [26], [30]. The non-classic monocyte subset (CD14+CD16++) produces pro-inflammatory cytokines and plays a role in cardiovascular disease [27], [28].

**Figure 1:**
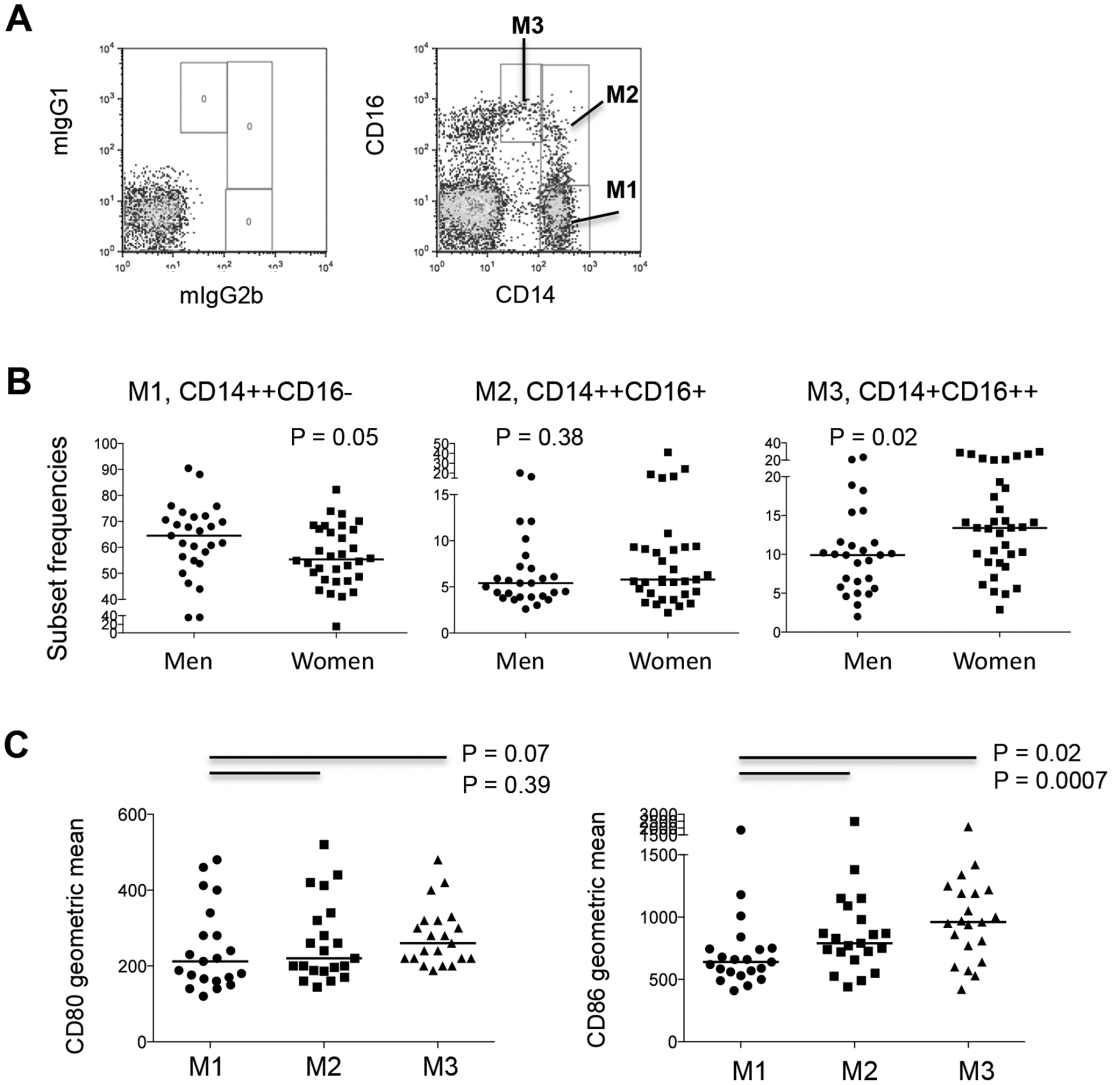
Increased percentage of the non-classic monocyte subset in total monocytes in control women compared to control men. (A) Dot plots of gating strategy for monocyte subsets. (B) Percentage (median) of classic (M1, CD14++CD16-), intermediate (M2, CD14+CD16+) and non-classic (M3, CD14+CD16++) monocyte subsets in women and men in fresh blood-samples. (C) Geometric mean of CD80 and CD86 expression among 3 subsets of monocytes tested in fresh blood-samples.

To focus on the gender differences in monocyte activation *in vivo* and to avoid monocyte activation *in vitro*, we analyzed cells in fresh blood samples from 27 healthy male controls, 32 healthy female controls, and 25 female patients with SLE. Monocytes and their subsets were identified by the expression of CD14 and CD16. The gating strategy for the 3 monocyte subsets is shown in Fig. 1A. Representative dot plots from one control donor (Fig. 1A) and summary figures (Fig. 1B) from 27 healthy men and 32 healthy women are shown. The percentage of non-classic monocytes (CD14+CD16++) was significantly increased in healthy women (median [IQR], 13.4% [9.0%–19.3%]) compared with healthy men (median [IQR], 9.9% [5.8%–11.6%]). The percentage of classic (CD14++CD16-) monocytes was significantly decreased in healthy women (median [IQR], 55.4% [47.9%–67%]) compared with healthy men (median [IQR], 64.5% [54.9%–71.7%]). The percentage of intermediate (CD14++CD16+) monocytes was similar between men and women (Fig. 1B).

Next, to assess whether the 3 monocyte subsets represent different activation stages, we compared the expression of monocyte activation markers CD80 and CD86 on the 3 subsets in fresh whole-blood samples. Of interest, there was a gradual increase in CD86 expression and a trend towards increased CD80 expression from classic to intermediate to non-classic monocytes, implying the stages of monocyte activation and differentiation are linked (Fig. 1C).

### Soluble CD14 is induced in activated monocytes by LPS *in vitro*, and is increased in plasma from healthy women compared to healthy men

**Figure 2:**
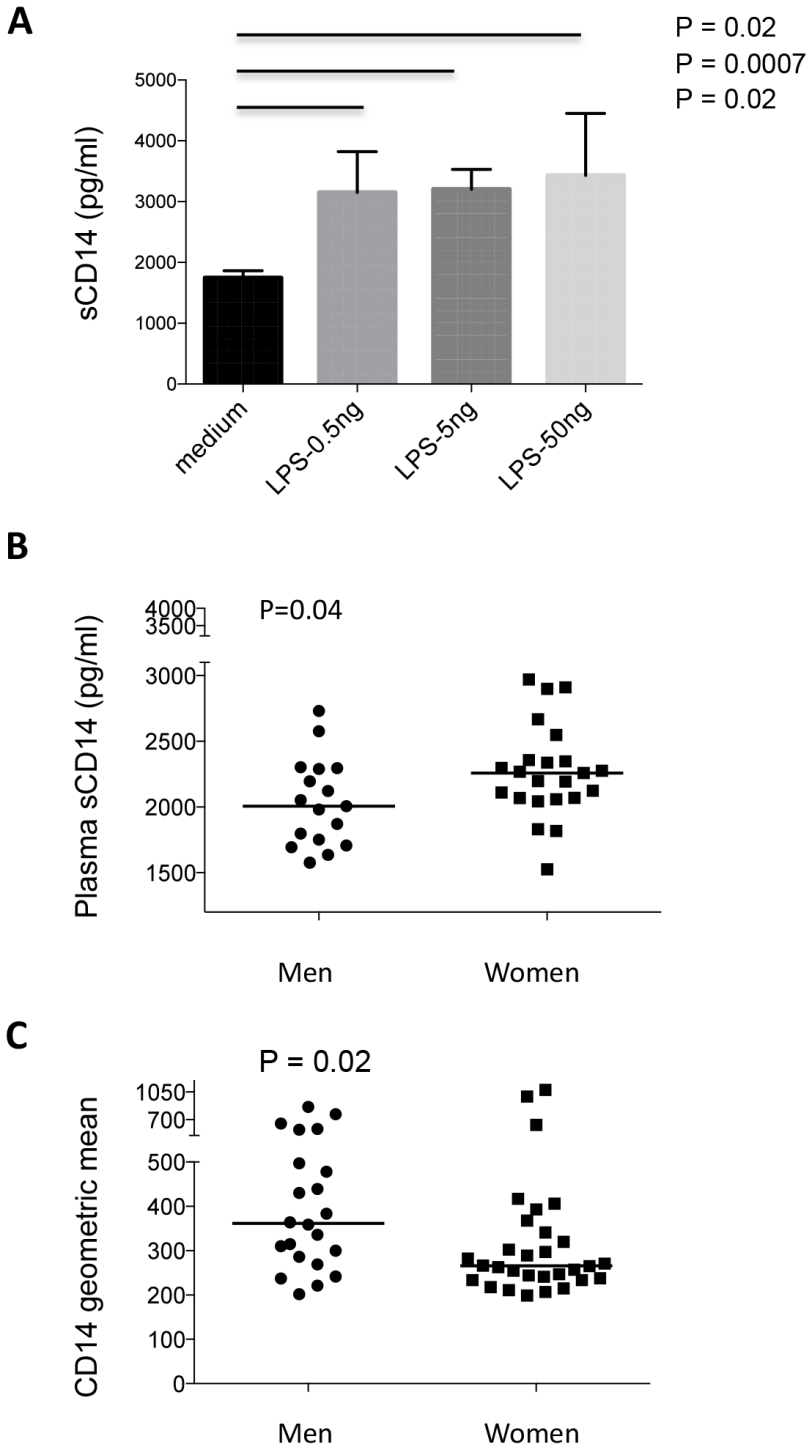
Monocytes produce sCD14 in cell culture supernatants in response to LPS; Plasma levels of sCD14 are increased in control women compared to men. (A) LPS from *E coli* has the ability to stimulate purified monocytes to produce sCD14 in cell supernatants after overnight culture. Soluble CD14 was tested by ELISA, n = 5. (B) Plasma levels of sCD14 (pg/mL) were measured by ELISA in healthy women and men. (C) Geometric mean expression of CD14 on monocytes was analyzed in healthy women and men by flow cytometry.

To investigate monocyte activation *in vivo* versus *in vitro*, total monocytes were purified by negative selection. Monocytes were cultured with 3 different concentrations of LPS (0.5, 5, 50 ng/mL) for 5 hours. Cell culture supernatants were harvested to assay for sCD14. All 3 concentrations of LPS induced significant production of sCD14 in cell culture supernatants compared to medium controls (Fig. 2A). Monocyte activation was then analyzed by plasma levels of sCD14 *in vivo*. Consistent with the results in Fig. 1; healthy women exhibited elevated levels of plasma sCD14 (Fig. 2B, median [IQR], 2258 pg/mL [2068 pg/mL–2358 pg/mL]) compared with healthy men (2006 pg/mL [1729 pg/mL–2292 pg/mL], and lower levels of membrane CD14 expression (Fig. 2C, median [IQR], 361.5 [281.8–516.3] and 265.5 [237–347.8] for men and women respectively). These results suggest that monocytes are more activated *in vivo* in healthy women compared to healthy men.

### Patients with SLE exhibit further monocyte activation compared to healthy women

**Figure 3:**
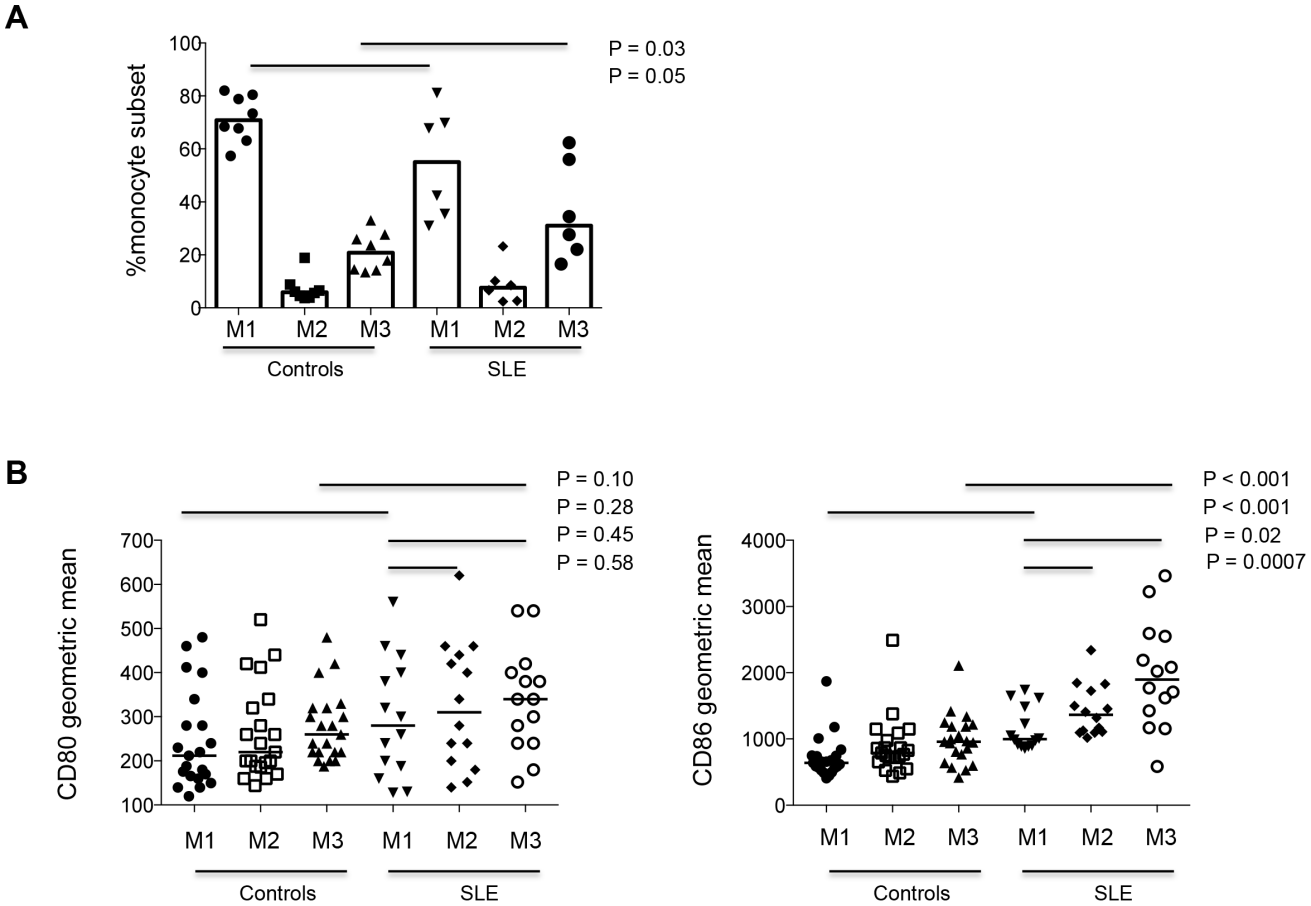
Female patients with SLE have further elevated proportions of non-classic monocytes and further reduced proportions of classic monocytes compared to female controls. (A) Fresh blood-samples were tested for the proportions of three monocyte subsets (CD14++CD16-, CD14++CD16+ and CD14+CD16++) in total monocyte population in 8 female controls and 6 female patients with SLE. (B) Geometric mean expression of CD80 and CD86 on monocytes was tested in female controls and female patients with SLE.

Prior studies showed that non-classic monocytes produce pro-inflammatory cytokines and may contribute to autoimmune diseases [26]. We asked the question whether the sex differences in monocyte activation seen in Fig. 1 and Fig. 2 would be accentuated comparing controls to SLE patients. The three subsets of monocytes were assessed in fresh whole-blood samples from healthy control women and women with SLE matched for age and race. Consistent with the finding in healthy women versus healthy men, patients with SLE have further skewed proportions of monocyte subsets. As shown in Fig. 3A, the percentage of non-classic monocytes (CD14+CD16++) in PBMCs was significantly increased in women with SLE compared to healthy women (median [IQR], 20.79% [14.32%–27.26%] and 31.06% [20.67%–57.59%] for controls and patients respectively). The percentage of classic (CD14++CD16-) monocytes was significantly decreased in women with SLE compared with healthy women (median [IQR], 70.89% [64.29%–80.07%] and 55.08% [34.35%–72.59%] for controls and patients respectively). The percentage of intermediate (CD14++CD16+) monocytes was similar between healthy women and women with SLE (Fig. 3A).

Next, we compared the expression of monocyte activation markers CD80 and CD86 on the 3 subsets in fresh whole-blood samples. As shown in controls, monocytes showed gradual increases in CD86 expression between subsets, but even higher levels of CD80 and CD86 was present on each monocyte subsets in patients with SLE compared to controls (Fig. 3B). These findings imply enhanced *in vivo* activation and differentiation of monocytes in SLE.

### Increased total monocytes, increased percentage of IL-6 producing monocytes and increased levels of plasma sCD14 in women with SLE compared to healthy controls

**Figure 4:**
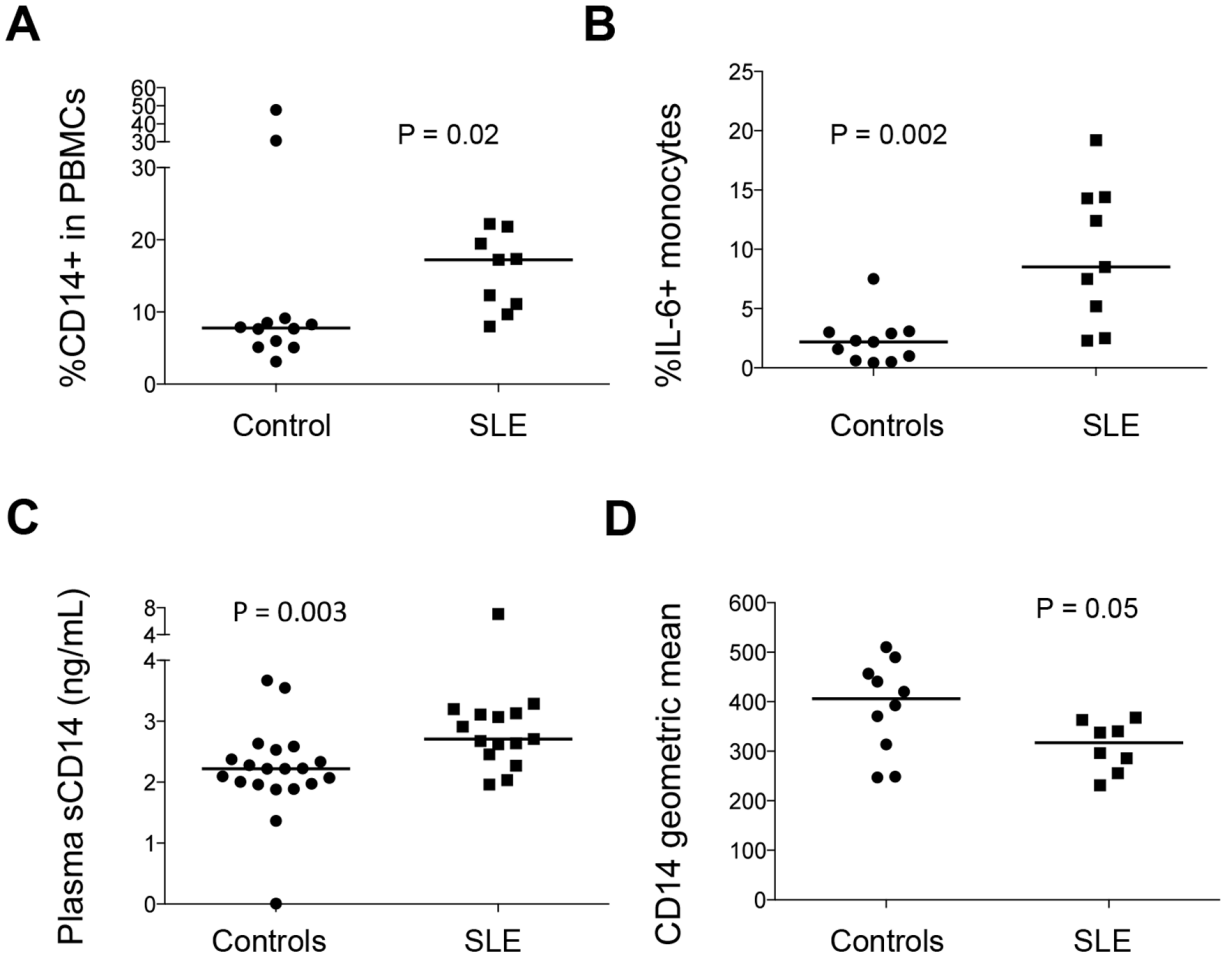
Female patients with SLE have higher levels of plasma sCD14, higher proportions of total monocytes and IL-6-producing monocytes compared to female controls *ex vivo*. (A) Blood was tested for the proportions of CD14+ monocytes in PBMCs in women in 12 controls and 9 patients with SLE. (B) Blood was tested for the percentage of IL-6-producing monocytes among total monocytes by intracellular staining by flow cytometry in 11 controls and 9 patients. (C) Plasma levels of sCD14 were tested by ELISA (ng/mL) in 10 controls and 15 patients. (D) Geometric mean expression of CD14 on monocytes was tested in 10 controls and 8 patients. All donors were women.

To further study monocyte activation *in vivo* in patients with SLE, the percentage of total PBMCs that are monocytes (CD14+), the percentage of IL-6-producing monocytes in total monocytes, and plasma levels of sCD14 were tested in healthy female controls and female patients with SLE. We found that patients had higher percent total monocytes (Fig. 4A, median [IQR], 17.23% [10.4%–20.66%] and 7.79% [5.34%–8.98%] for patients and controls respectively), higher percentage of IL-6-producing monocytes (Fig. 4B, median [IQR], 2.2% [0.61%–3.0%] and 8.5% [3.85%–14.35%] for patients and controls respectively), and higher plasma levels of sCD14 compared to female controls (Fig. 4C, median [IQR], 2707 pg/mL [2456 pg/mL–3129 pg/mL] and 2222 pg/mL [1963 pg/mL–2492 pg/mL] for patients and controls respectively). We also found that CD14 membrane expression on monocytes was further reduced compared to the levels in female controls (Fig. 4D, median [IQR], 317 [263.4–357.6] and 406.5 [297.7–465.1] for patients and controls respectively). These results suggest that the sex differences in monocyte activation *in vivo* are further enhanced comparing controls and patients with SLE.

### Plasma levels of IL-6 are positively associated with serum creatinine and plasma levels of sCD14

**Figure 5:**
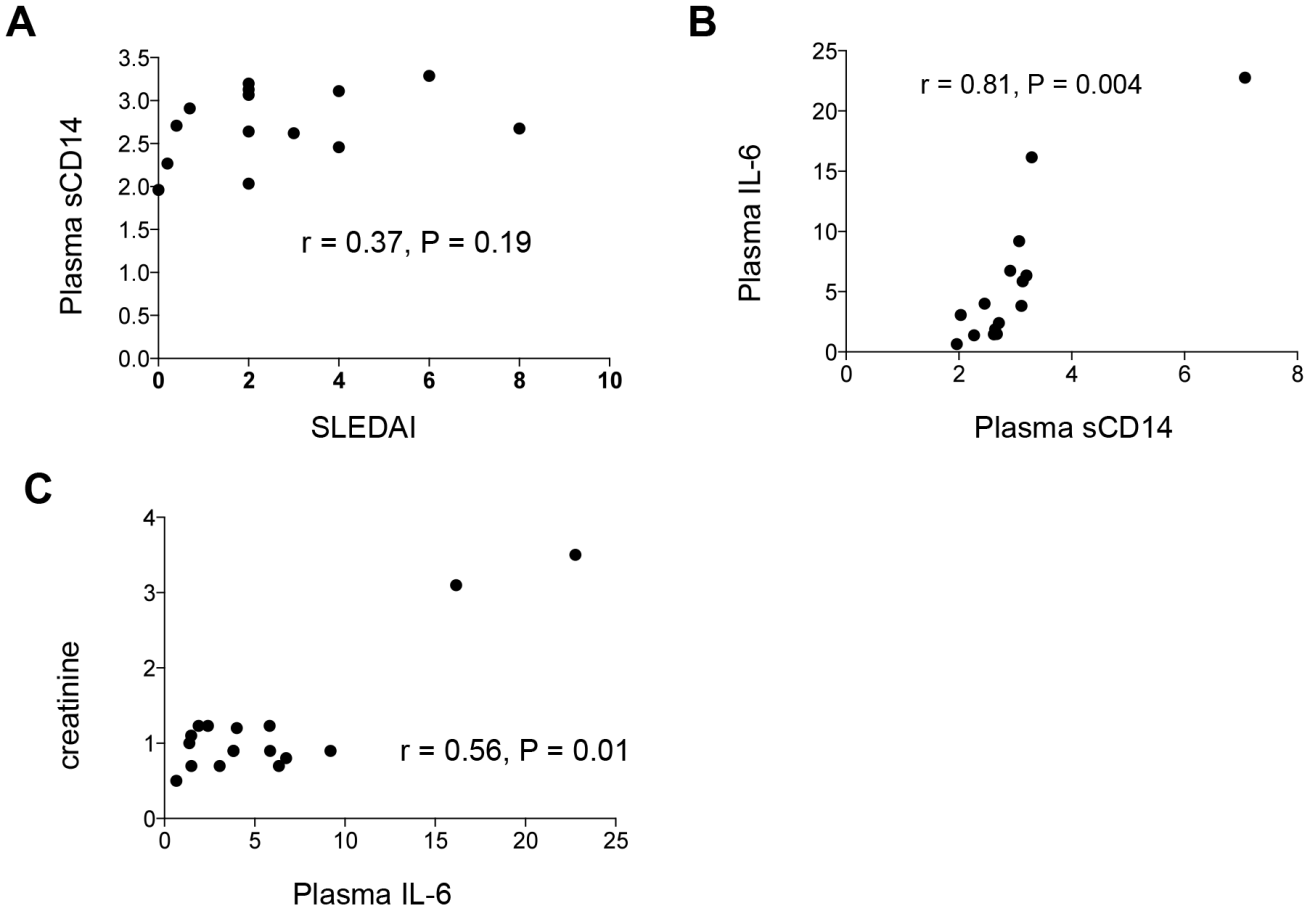
Plasma levels of IL-6 are positively correlated to serum creatinine and plasma levels of sCD14 in patients with SLE. (A) Correlation between plasma levels of sCD14 and SLEDAI scores (B) Correlation between plasma levels of IL-6 and serum levels of creatinine. N = 16. (C) Correlation between plasma levels of IL-6 and sCD14. N = 14. Spearmen correlation test.

To investigate the associations of monocyte activation with SLE disease progression, we assessed the correlations between monocyte activation markers and clinical tests of lupus disease activity, including serum creatinine, serum C3 and C4, the SLE disease Activity Index (SLEDAI), and the disease damage index (SLICC DI). We found there was a trend but did not achieve significance between plasma level of sCD14 and SLEDAI score (Fig. 5A, r = 0.37, P = 0.19). IL-6 can be released by activated monocytes and plays a role in SLE disease pathogenesis [52], [53]. Therefore the correlation between plasma IL-6 and another marker of monocyte activation, plasma sCD14 was analyzed. Indeed, there was a direct relationship between plasma sCD14 and plasma IL-6 (Fig. 5B, r = 0.81, P = 0.004). Furthermore, we found that plasma levels of IL-6 were positively correlated with serum creatine (Fig. 5C, r = 0.56, P = 0.01). These results suggest that plasma IL-6 and sCD14 are impacted by similar mechanisms *in vivo*; we assume they were both produced by activated monocytes. The correlation between plasma levels of IL-6 and serum creatinine suggest that IL-6 may play a role in kidney damage in SLE disease or that plasma IL6 was not cleared due to decreased renal function.

There was no correlation between any of the other lab or clinical parameters with monocytes subsets or IL6. The patients were on a variety of medications including prednisone, plaquenil and immunosuppressants and there is no correlation between medications and the measures we report.

## Discussion

The effect of sex hormones on monocyte activation and TLR4 responsiveness can be mediated through an indirect or direct pathway. The direct pathway involves signaling through the sex hormone receptors such as ERα that either impact cytoplasmic kinase pathways or via ERα mediating gene transcription. The indirect pathway can be mediated by the sex hormones impacting gastrointestinal microbial product translocation or by impacting clearance of microbial products. The effects of estrogen on monocyte activation and TLR4 responses may also include modulation of TLR4 expression, TLR4 signaling pathways, LPS interaction cofactors (e.g., TLR4, CD14, MD2 and LBP), and levels of TLR4 ligands. As a consequence of enhanced monocyte activation and TLR4 responsiveness, women have overall higher levels of inflammatory markers and altered proportions of monocyte subsets compared to men. These sex differences in monocytes are further accentuated comparing SLE patients with female controls, and may partially account for sex bias in the prevalence of SLE disease.

Consistent with our results, previous studies showed that patients with SLE have elevated monocyte number compared to controls, that monocytes from SLE patients spontaneously produce IL-6 [54], [55], and that LPS and TLR4 responses play a role in kidney damage in SLE [56]–[61]. Moreover, plasma levels of sCD14, secreted by monocytes in response to LPS, are elevated, and the expression of membrane CD14 on monocytes are reduced in patients with SLE compared to controls [62]. These studies are consistent with our results and indicate that monocytes are activated *in vivo*, produce pro-inflammatory cytokines (e.g., IL-6), and contribute to chronic inflammation in SLE disease [63]. Therefore, there may be a link between monocyte activation, TLR4 signaling pathway, monocyte maturation and differentiation, and SLE disease pathogenesis.

In the current project, we first report that healthy women have roughly 3.5% higher percentage of non-classic monocytes and 9.1% lower percentage of classic monocytes compared to men. Furthermore, this subset of non-classic (CD14+CD16++) monocytes is expanded in sepsis patients, and also in SLE [31], [34]. This subset of monocytes is also a major source of pro-inflammatory cytokines (TNF-α and IL-1β) induced in response to TLR7/8 ligands. In contrast, classic monocytes produce IL-10 in response to the TLR4 ligand LPS [26]. Therefore an increase in the pro-inflammatory monocyte subset and a decreased classic monocyte subset may cooperate to drive elevated levels of persistent immune activation in women compared to men, and chronic inflammation in patients with SLE compared to controls. Treatments directed against TLR4 signaling down-stream pro-inflammatory cytokines (e.g., TNF-α, IL-6 and IL-1β) are partially effective in SLE disease [64]–[67]. Therefore, we believe, based on our data that treatment should not only target TLR7/8 and TLR9, but also should target the effect of TLR4 signaling on lupus pathogenesis, and gender differences in other autoimmune diseases.

It appears that two patient outliers had highest frequencies of non-classical monocytes and plasma levels of sCD14. The clinical characteristics of these two outliers are shown in table 1. These two outliers also had high levels of urine protein and creatinine. After removing two patient outliers, there were no differences in the frequencies of monocyte subsets in control women and lupus women (data not shown). However, the geometric mean expression of CD86 in non-classic monocyte subsets was still higher in lupus patients than controls (P = 0.005, data not shown) after removing two outliers of patients, indicating that monocytes are activated *in vivo* in patients compared to controls. Furthermore, after removing two patient outliers, there was still a correlation between plasma IL-6 and plasma sCD14 (r = 0.71, P = 0.008, data not shown), but there was no correlation between urine creatinine and plasma IL-6 (r = −0.02, P = 0.89, data not shown). The consistency of monocyte activation (frequencies of non-classic monocytes and plasma sCD14) and kidney damage (urine levels of protein and creatinine) suggests that monocyte activation contributes to disease pathogenesis.

**Table 1:** Characteristics of the 2 patient outliers.

	Patient#3019	Patient#3127
Age	41	45
Gender	Female	Female
Medicine	AZATHIOPRINE+PREDNISONE	AZATHIOPRINE
Serum C3, mg/dl	76.4	122
Serum C4, mg/dl	17.6	24.6
Months of SLE diagnosis	4	12
Urine protein, mg/dl	300	300
Urine creatinine, mg/dl	86.2	64.7
SLEDAI score	6	0

SLEDAI  =  SLE Disease Activity Index.

As noted above, prior studies demonstrated enhanced responsiveness to TLR7 ligands of peripheral blood mononuclear cells and dendritic cells from women compared to men as measured by IFNα production. The mechanisms for this enhanced responsiveness is unknown but does appear to be estrogen sensitive and requires the presence of ERα. We previously demonstrated that the response of dendritic cells and B cells to TLR ligands in mice is significantly dampened in the absence of ERα. Furthermore, in the absence of ERα, lupus like disease in three different mouse strains was protective against the development of kidney disease with no effect on autoantibody production. These findings led us to investigate the role of sex hormone signaling on innate immune responses and to translate our murine findings into human studies.

The exact mechanisms by which the enhanced activation of monocytes and dendritic cells in women compared to men is unclear but does appear to be indirect. This is perhaps best illustrated by studies done comparing TLR responsiveness of dendritic cells from postmenopausal women, premenopausal women and men. Dendritic cells from premenopausal women were more responsive than DCs from men. Surprisingly, however, DCs from postmenopausal women were significantly less responsive to TLR ligands than DCs from premenopausal women [16]. When the postmenopausal women were placed on hormone replacement therapy and then retested, their DCs had enhanced responsiveness similar to that of premenopausal women [16]. Adding estrogen *in vitro* to cultured DCs from postmenopausal women had no effect suggesting that the enhanced responsiveness of DCs from premenopausal women was not a direct effect of estrogen on the cells, but due to an indirect effect of estrogen *in vivo*. Estrogen has multiple biologic effects as almost all cells express estrogen receptors. Thus the mechanism may involve effects on the GI tract, liver clearance, expression of TLR ligands, and/or developmental effects during the maturation of immune cells. Why lupus patients have further accentuation of these responses is unclear at present.

The strengths of this study are the demonstration of the effects of sex on monocyte subsets and monocyte activation. We are unable at this time to identify the underlying mechanism for this effect. In summary, these results suggest that monocytes from women compared to men and SLE compared to controls are activated, release pro-inflammatory cytokines, and may contribute to SLE disease pathogenesis.

## Supporting Information

S1 File **The clinical characteristics of participants.**
(XLS)
